# Regulatory microRNAs in Brown, Brite and White Adipose Tissue

**DOI:** 10.3390/cells9112489

**Published:** 2020-11-16

**Authors:** Seley Gharanei, Kiran Shabir, James E. Brown, Martin O. Weickert, Thomas M. Barber, Ioannis Kyrou, Harpal S. Randeva

**Affiliations:** 1 Warwickshire Institute for the Study of Diabetes, Endocrinology and Metabolism (WISDEM), University Hospitals Coventry and Warwickshire NHS Trust, Coventry CV2 2DX, UK; s.gharanei@warwick.ac.uk (S.G.); martin.weickert@uhcw.nhs.uk (M.O.W.); t.barber@warwick.ac.uk (T.M.B.); i.kyrou@aston.ac.uk (I.K.); 2 Warwick Medical School, University of Warwick, Coventry CV4 7AL, UK; 3 Aston Medical Research Institute, Aston Medical School, College of Health and Life Sciences, Aston University, Birmingham B4 7ET, UK; shabirk@aston.ac.uk (K.S.); J.E.P.BROWN@aston.ac.uk (J.E.B.); 4 School of Biosciences, College of Health and Life Sciences, Aston University, Birmingham B4 7ET, UK; 5 Centre of Applied Biological & Exercise Sciences, Faculty of Health & Life Sciences, Coventry University, Coventry CV1 5FB, UK

**Keywords:** adipose tissue, BAT, WAT, brite, beige, adipocytes, micro-RNA, metabolism, energy homeostasis, obesity, diabetes

## Abstract

MicroRNAs (miRNAs) constitute a class of short noncoding RNAs which regulate gene expression by targeting messenger RNA, inducing translational repression and messenger RNA degradation. This regulation of gene expression by miRNAs in adipose tissue (AT) can impact on the regulation of metabolism and energy homeostasis, particularly considering the different types of adipocytes which exist in mammals, i.e., white adipocytes (white AT; WAT), brown adipocytes (brown AT; BAT), and inducible brown adipocytes in WAT (beige or brite or brown-in-white adipocytes). Indeed, an increasing number of miRNAs has been identified to regulate key signaling pathways of adipogenesis in BAT, brite AT, and WAT by acting on transcription factors that promote or inhibit adipocyte differentiation. For example, MiR-328, MiR-378, MiR-30b/c, MiR-455, MiR-32, and MiR-193b-365 activate brown adipogenesis, whereas MiR-34a, MiR-133, MiR-155, and MiR-27b are brown adipogenesis inhibitors. Given that WAT mainly stores energy as lipids, whilst BAT mainly dissipates energy as heat, clarifying the effects of miRNAs in different types of AT has recently attracted significant research interest, aiming to also develop novel miRNA-based therapies against obesity, diabetes, and other obesity-related diseases. Therefore, this review presents an up-to-date comprehensive overview of the role of key regulatory miRNAs in BAT, brite AT, and WAT.

## 1. Introduction

Adipose tissue (AT) consists of different cell types (e.g., mature adipocytes, pre-adipocytes, and immune cells) and secretes more than 600 different molecules collectively referred to as adipokines, functioning as an endocrine organ with pleiotropic functions that regulate whole body metabolism [1,2,3]. Based on morphology and function, AT has been traditionally categorized into white and brown subtypes (WAT and BAT, respectively), whilst brite AT (beige or brown-in-white AT) has more recently attracted research attention due to its potential role in combating obesity and obesity-related diabetes [4,5,6,7].

AT originates from the mesoderm, where WAT and BAT follow separate differentiating lineages, which are tightly orchestrated by an array of transcription factors [8,9]. Of note, mesenchymal stem cells can commit to adipogenic or myogenic lineages that give rise to white or brown adipocytes or myocytes [10]. As such, BAT and myocytes develop from the myogenic lineage, deriving from myogenic factor 5 positive cells (MYF5^+^), whereas WAT has an adipogenic lineage and is MYF5^−^ [11].

### 1.1. White Adipose Tissue (WAT)

WAT is markedly heterogeneous, consisting mainly of mature adipocytes and the stromal vascular fraction (SVF) [2]. The latter contains various cell types, including immune cells and pre-adipocytes, which are the major adipocyte progenitor cells [2]. The primary physiological role of WAT is to store energy in the form of lipids, with excess free fatty acids (FFA) and glycerol being stored as triacylglycerols (triglycerides) in adipocytes. When energy is required by other organs/tissues, these lipids are hydrolyzed and FFA are released back into the circulation [12]. However, it is now well-established that WAT has multiple metabolic, endocrine and immune functions [13], which regulate a number of physiological processes (e.g., energy and glucose homeostasis, appetite, reproduction and vascular functions) through secretion of adipokines [14,15]. Obesity and dysregulation of normal WAT function can lead to accumulation of lipids in ectopic sites/tissues, promoting the development of cardio-metabolic diseases [14,15]. Each mature WAT adipocyte has a single, large lipid droplet that occupies most of the space within the cytoplasm, while the rest of its components (e.g., the nucleus, mitochondria and endoplasmic reticulum) are pushed towards the sides of the cell [16,17].

Of note, WAT can be divided into two main categories, namely, visceral and subcutaneous (scAT) with each having distinct functions in health and disease [2]. Visceral AT constitutes a smaller percentage of the total body fat mass and is found in the intra-abdominal regions (mainly in the omentum and mesentery), and around major organs, such as the kidneys, liver and heart [18]. Conversely, scAT accounts for most of the total body fat mass and is mainly located in the abdominal wall and the femoral-gluteal areas [2,16,19]. Based on location, AT can be further categorized into different depots. For example, in the mouse visceral AT is categorized into peri-gonadal, peri-renal, triceps-associated and cardiac, while scAT into anterior, inter-scapular and inguinal [2]. The exact range of functions of each AT depot and their response to different stimuli (e.g., nutritional and hormonal stimuli) is yet to be fully clarified, including potential differences between species [20].

WAT expansion, dysfunction, and inflammation are hallmarks of obesity and play a critical role in the development of highly prevalent cardio-metabolic disorders, such as insulin resistance (IR), type 2 diabetes mellitus (T2DM), non-alcoholic fatty liver disease (NAFLD), atherosclerosis, and cardiovascular disease (CVD) [21]. Indeed, compelling research indicates that accumulation of excess ectopic and visceral AT are key contributors to the pathophysiology of these obesity-related cardio-metabolic diseases due to their metabolically deleterious profile/function in obesity [22,23,24].

### 1.2. Brown Adipose Tissue (BAT)

BAT is distinctively thermogenic, regulating body temperature by expending energy derived from lipids to produce heat, whilst it also has an active secretory role by releasing adipokines and factors with paracrine and/or endocrine actions [25,26]. In mice, BAT is located in five major depots, i.e., inter-scapular, mediastinal, peri-renal, axillary, and cervical BAT [8,27]. In humans, until recently BAT was thought to be functional only in neonates; however, it is now well-documented that adult humans also have certain BAT depots which can be activated by cold [28,29,30]. BAT is characterized by having multi-locular small lipid droplets and a high number of iron containing mitochondria, which account for its characteristic brown color [25,26]. Uncoupling protein 1 (UCP1) is exclusively expressed within the inner membrane of the mitochondria of brown adipocytes and uncouples fuel oxidative phosphorylation from adenosine triphosphate (ATP) synthesis to generate heat, thus playing a key role in energy dissipation via non-shivering thermogenesis [8,27]. Notably, the thermogenic program of BAT is activated not only by cold exposure but also by exercise and other factors, such as adrenergic stimulation, natriuretic peptides, retinoids, capsinoids, thyroid hormones, and glucocorticoids [31].

Overall, BAT is highly innervated by the sympathetic nervous system (SNS) and so, upon cold exposure or overfeeding, BAT thermogenesis is stimulated by the release of norepinephrine which activates the β3 adrenergic receptors expressed on brown adipocyte cell membranes [32]. In turn, this activation results in stimulation of intercellular cyclic adenosine 3′,5′-monophosphate (cAMP) synthesis that activates protein kinase cAMP dependent (PKA) signaling [27]. This further stimulates lipid catabolism (lipolysis of triglycerides) to release FFA that are used as a fuel substrate for UCP1 derived thermogenesis [27]. Due to consuming energy from glucose and lipids for thermogenesis, BAT is regarded as a potential target for novel interventions against obesity [10,33]. Indeed, BAT activity increases the whole-body energy expenditure and, hence, may play a protective role against obesity and obesity-related complications [34,35,36]. Moreover, BAT activity appears to be impaired in individuals with obesity and correlates inversely to the body mass index (BMI), body fat content, and central adiposity [37,38].

### 1.3. Brite Adipose Tissue (Beige or Brown-in-White Adipose Tissue)

Brite adipocytes are found in WAT depots, where white adipocytes can be converted to brown-like adipocytes [10]. Brite AT is even more heterogeneous, as it originates from MYF5^+^ and MYF5^𢈒^ lineages [4,5,6,7,39,40], and shares morphological, molecular, and thermogenic characteristics/functions with typical BAT. As such, brite AT expresses inducible *UCP1* and exhibits a medium density of mitochondria [4,5,6,7]. The plasticity of WAT and brite AT appears to play a significant role [41], as bidirectional inter-conversion processes have been documented between WAT and brite AT [42]. In addition to expressing adipogenic genes (*UCP1*; Cell death-inducing DNA fragmentation factor-like effector A, *CIDEA*; CCAAT/enhancer-binding protein beta, *C/EBPB*; and Peroxisome proliferator-activated receptor gamma coactivator 1alpha, *PGC1A*), brite adipocytes also express certain brite markers, such as CD137 (4-1BB, or tumor necrosis factor receptor superfamily member 9, TNFRSF9), transmembrane protein 26 (TMEM26), or T-Box transcription factor 1 (TBX1) [36,43]. Interestingly, browning of WAT is induced by cold exposure or genetic modification in a process whereby the expression of genes involved in BAT function are induced in WAT, thus resulting in brite (“bright” or “beige”) adipocytes [36,43]. This WAT browning is achieved by key transcription factors, including PR domain-containing protein 16 (PRDM16) and peroxisome proliferator-activated receptors (PPARs) [5,44]. Indeed, PPARA (PPAR-alpha) activation promotes beige adipogenesis via PRDM16 and PGC1A [5,6]. Moreover, PPARG (PPAR-gamma) is indispensable for both WAT and BAT formation [45,46], and PPARG activators/agonists may be used to induce browning of WAT [11,47]. Typical BAT develops much earlier in life than WAT but both share similar transcription cascades which involve PPARG and CCAAT/enhancer-binding proteins (C/EBPs) [48]. Of note, fully functional brite adipocytes have been found in both infants and adults on cold exposure [49]. As will be detailed in the following sections, certain microRNAs regulate BAT and WAT function and differentiation [50,51,52]. Such microRNAs regulating white, brown, and brite adipogenesis often target key transcription factors (e.g., PRDM16; PPARG; C/EBPB; PGC1A/B; and Early B-cell factor transcription factor 2, EBF2), thus, elucidating their specific adipogenic role and functions is emerging as an important aspect of AT biology.

### 1.4. Transcription Factors and Browning Agents

PPARs represent a group of nuclear proteins which function as transcription factors [53]. Two members of this family, i.e., PPARA and PPARG, play a pivotal role in AT development and differentiation [11]. As aforementioned, *PPARG* is highly expressed in both WAT and BAT [11] and constitutes the master transcription factor in the differentiation program of BAT. As such, PPARG has a crucial role for tissue development, function and survival, inducing *UCP1* expression during adipogenesis [11]. Conversely, PPARA induces *PGC1A*, with *PPARA* ablation decreasing BAT specific genes (e.g., Zic family member 1, LIM homeobox 8, and *PRDM16*) [54].

PRDM16 is a zinc-finger protein which controls the switch between skeletal myoblasts and brown adipocytes [44], and stimulates adipogenesis by directly binding to *PPARG* [44]. PRDM16 also binds to and co-regulates many other regulators (e.g., PGC1A; PGC1B; C/EBPs; EBF2; and Euchromatic Histone Lysine Methyltransferase 1, EHMT1) to promote brown adipocyte specific gene induction [54]. Thus, PRDM16 configures a transcriptional complex with C/EBPB which controls the cell fate switch from MYF5^+^ cells to brown pre-adipocytes and activates *PPARG* expression, inducing the thermogenic program [55]. PRDM16 is essential for embryonic BAT development. However, recent studies have shown that BAT is not affected in *Prdm16* knockdown mice, suggesting that other factors also mediate these functions [55]. Notably, PRDM16 appears to be required for the maintenance of BAT function during aging in mice [56].

C/EBPs are transcription factors which control the differentiation of a range of cell types and gene expression in early adipogenesis [57]. C/EBPB and C/EBPD regulate the expression of *C/EBPA* and *PPARG* involved in the last stage of adipogenic differentiation [57]. In addition, C/EBPA functions to maintain *PPARG* expression and cooperatively regulates gene transcription to promote and maintain the differentiation of adipocytes, insulin sensitivity, and glucose and lipid metabolism [58].

PGC1A is a cold inducible co-activator of PPARA and PPARG and a key regulator of mitochondrial biogenesis, adaptive thermogenesis, and oxidative metabolism [59]. Conversely, the transcription repressor receptor–interacting protein 140 (RIP140) inhibits adipogenesis and is able to block the effects of PGC1A [60]. Notably, mice devoid of RIP140 are lean and have increased oxygen consumption, whilst being resistant to obesity induced by high-fat diet (HFD) [60]. Moreover, EBF2 is an essential mediator of brown adipocyte commitment and terminal differentiation, playing a crucial role in adequate PPARG binding to *UCP1* [61].

### 1.5. MicroRNA Function and Regulatory Mechanisms

MicroRNAs (miRNAs) constitute a class of short (20–24 nucleotide) non-coding, single stranded RNAs that regulate gene expression at the post transcriptional level by binding to the mRNA of the target gene [62]. Following their discovery in the 1990s [63], miRNAs have been shown to regulate a wide spectrum of biological processes and are implicated in metabolism and the pathophysiology of multiple diseases whilst may also serve as corresponding diagnostic biomarkers [64,65,66,67]. Indeed, miRNAs are now regarded to play a key role in AT biology, implicated in regulating the differentiation and function of both WAT and BAT [50,51,52].

**Figure 1:**
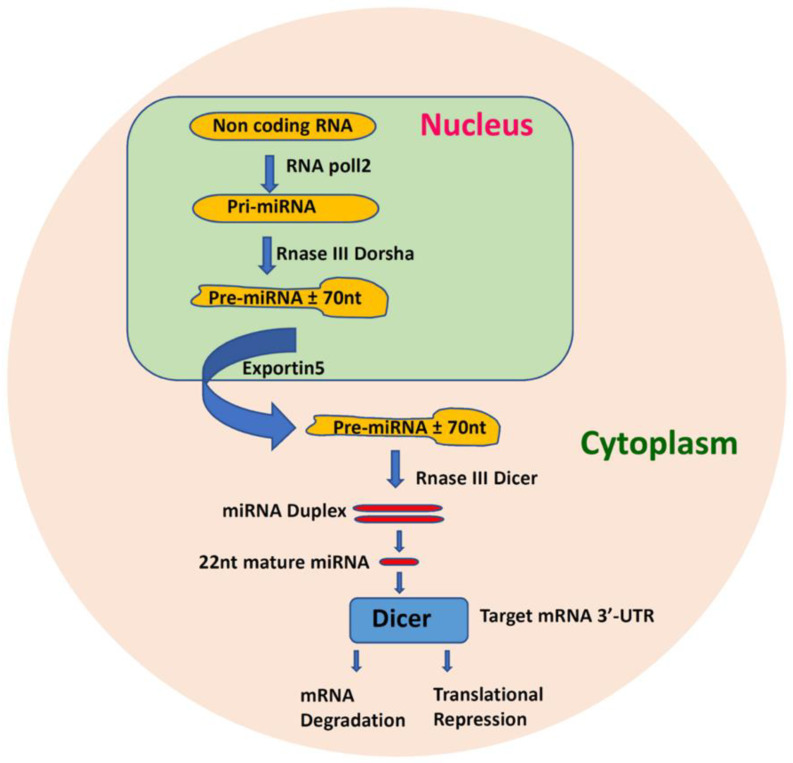
Brief schematic representation of the microRNA (miRNA) processing pathway. The primary transcripts of miRNAs, called pri-miRNAs, are transcribed by RNA polymerase II. The RNase III enzyme Drosha further processes the pri-miRNAs into hairpin shape precursors of 70 to 100 nucleotides (nt) called pre-miRNA, which are then exported to the cytoplasm by exportin-5 for further processing. In the cytoplasm, another RNase III enzyme, Dicer, cleaves the pre-miRNAs to form linear double stranded intermediate miRNA called miRNA duplex. The ∼22-nucleotide long mature miRNAs strand obtained from the intermediate duplex is then loaded into the RNA-induced silencing complex (RISC) to silence the target messenger RNA (mRNA) by destabilizing it and/or inhibiting its translation.

MiRNAs function by binding to the RNA-induced silencing complex (RISC), and through this by subsequently binding to the 3’ untranslated regions (3’ UTR) of mRNAs causing translational repression or mRNA degradation [68,69]. Initially, miRNAs are transcribed by RNA-polymerase II as 5′-capped polyadenylated precursors which are known as primary miRNA (pri-miRNA) [70,71]. After transcription, this pri-miRNA undergoes maturation by Drosha which releases a small hairpin shaped RNA in the nucleus, called pre-miRNA [70]. In turn, pre-miRNA is exported to the cytoplasm by exportin-5 and is cleaved by DICER to form a small RNA duplex (Figure 1) [72]. Two mature miRNA species may be generated from the 3′ end (passenger strand) and the 5′ end (leading strand) of a pre-miRNA precursor. Only one species remains viable in most cases, whilst the complementary species is degraded; however, co-existence of miRNA-5p and -3p species is increasingly being reported [73].

MiRNAs are also found in blood, urine, and extracellular fluid, packaged inside lipid or lipid protein complexes, such as microvesicles, exosomes, or apoptotic bodies [74,75]. Specific miRNAs tend to be preferentially released by cells and, thus, are more prone to be found in the circulation [76]. Of note, AT-specific disruption of miRNA processing by knockdown of DICER and DiGeorge syndrome critical region 8 (DGCR8) has been shown to alter WAT accumulation and promote whitening of BAT, leading to impaired metabolic function [77,78,79]. Moreover, Mori et al. reported whitening of inter-scapular BAT in mice with a fat-specific DICER knockdown [78], while MiR-365, MiR-346 and MiR-362 rescued the whitening phenotype in DICER knockout mice [79]. Such studies provide evidence that supports an important role that miRNAs play in the functioning of both WAT and BAT, as detailed in the following sections. Intriguingly, the study by Thomou et al. showed that AT is a major source of circulating exosomal miRNAs and that mice with an AT-specific DICER knockout (ADicerKO) have reduced levels of circulating exosomal miRNA [75]. This study further showed that transplantation of WAT and BAT into ADicerKO mice restores the circulating levels of various miRNAs which are related with glucose tolerance improvement and decreased hepatic *Fgf21* mRNA and circulating FGF21 levels [75]. This further suggests that AT-derived circulating miRNAs can impact on the regulation of gene expression in distant tissues, thus acting as another form of adipokines [75].

## 2. MicroRNAs in BAT

**Figure 2:**
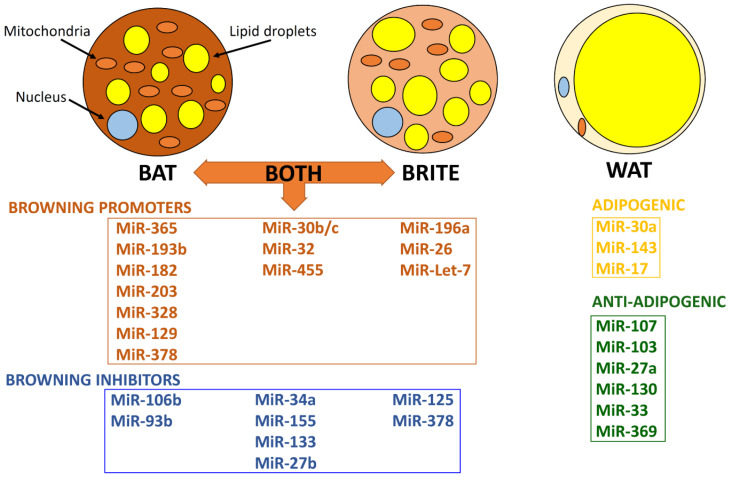
Key microRNAs (miRNAs) regulating brown adipose tissue (BAT), brite (beige or brown-in-white) adipose tissue, and white adipose tissue (WAT).

Certain miRNAs have been reported to specifically regulate BAT, including MiR-193b-365, MiR-182-203, MiR-106a-93, MiR-328, and MiR-129 [71,80]. These miRNAs target important thermogenic genes with either positive or negative impact on the BAT thermogenic program [71,80]. Moreover, various miRNAs, such as the MiR-193b-365 cluster, MiR-328, MiR-378, MiR-30b/c, MiR-455, and MiR-32, are activators of brown adipogenesis, whereas MiR-34a, MiR-133, MiR-155, and MiR-27b are brown adipogenesis inhibitors (Table 1 and Figure 2) [81,82,83,84]. Manipulation of these miRNAs has been shown to affect uncoupled respiration, glucose uptake, and thermogenesis, as well as whole-body energy expenditure, glucose tolerance, and insulin sensitivity [6,71,80]. Consequently, these miRNAs represent potentially important therapeutic targets for the treatment of obesity and related metabolic disorders, such as T2DM.

*MiR-193b-365:* MiR-193b and MiR-365 form a cluster that is located on chromosome 16 [85]. This cluster is highly expressed in BAT and is involved in the regulation of BAT differentiation, directly targeting *Runx1t1* (Runt-related transcription factor 1; translocated to, 1; which is an adipogenesis inhibitor) [85]. Moreover, the MiR-193b-365 cluster is positively regulated by PRMD16 [81]. Interestingly, Sun and colleagues reported that MiR-365 was sufficient to promote *Ucp1* expression in Dicer knockout mice [85]. However, in vivo studies showed that blocking this miRNA cluster does not change BAT morphology and the expression of BAT-related markers (UCP1 and PRMD16) [107]. Moreover, Oliverio et al. showed that MiR-193b regulates BAT differentiation by targeting a β-secretase (i.e., beta-site amyloid precursor protein cleaving enzyme 1; BACE1) which promotes myogenic differentiation [86]. This study also showed that loss of MiR-193 in brown adipocytes attenuated key thermogenic genes (*Cidea*, *Ucp1*, *Pgc1a* and *Prdm16*) [86]. It is therefore evident that this miRNA cluster is implicated in BAT regulation, but further studies are clearly required to clarify its exact function(s).

*MiR-182* and *MiR-203*: MiR-182 and MiR-203 are positive regulators of BAT adipogenesis, targeting *Insig1* (Insulin-induced gene 1) and *Pdgfr2* (Platelet-derived growth factor receptor 2) [77,81]. These BAT specific miRNAs are essential for the maintenance and differentiation of brown adipocytes in vivo [77]. Inhibition of MiR-182 or MiR-203 causes reduction in BAT adipogenic markers (e.g., UCP1, PGC1A, CIDEA) and mitochondrial markers (e.g., cytochrome c oxidase subunit 7), as well as downregulation of genes which control the respiratory electron transport and oxidative phosphorylation, whilst the complementary species is degraded [77]. When comparing the miRNA profiles in mouse and human BAT, Güller and colleagues found that MiR-182 and MiR-203 were part of the top ten miRNAs targeting the most genes involved in growth and development in mouse BAT; however, only MiR-203 was conserved in human BAT [108]. The absence of MiR-182 expression in human BAT might contribute to differences between mouse and human BAT development and function.

*MiR-106b-93:* MiR-106b and MiR-93 form a cluster that targets PPARA and acts as a negative regulator of BAT differentiation [109]. This cluster is a member of the MiR-17 family which controls stem cell differentiation in the developing mouse embryo [81]. MiR-106b-93 expression increases during brown adipocyte differentiation and in BAT under a HFD, but not in WAT [110]. Of note, MiR-106b-93 inhibition has been shown to induce brown adipogenic markers (e.g., UCP1, PRMD16, CIDEA, PPARA, PGC1A), as well as fatty acid-binding protein 4 (FABP4, also known as adipocyte protein 2, AP2), PPARG and adiponectin, and promote lipid droplet accumulation in brown adipocytes [109]. Interestingly, in mice, the expression of this cluster is increased in BAT by obesity [87], whilst overexpression of this cluster has been shown to suppress *Ucp1* expression [84]. Overall, this miRNA cluster is thought to play a significant role in energy homeostasis through negative regulation of brown adipocytes.

*MiR-328:* MiR-328 is a positive regulator of BAT differentiation which targets *Bace1*, with MiR-328 inhibition in brown adipocytes resulting in downregulation of thermogenic gene expression (*Cidea, Ucp1, Pgc1a* and *Prmd16*) [86,88]. Additionally, overexpression of this miRNA has been shown to instigate BAT differentiation and increase C/EBPB and UCP1 levels, as well as oxygen consumption rates in BAT, and can also impair muscle progenitor commitment [86]. Moreover, sequence similarities have been identified between MiR-193b and MiR-328, which appear to be co-expressed in most tissues, with enrichment in BAT and WAT and significant overlap between their target gene sets [86]. Overall, MiR-328 is considered to control brown adipogenesis by regulating the switch between muscle-specific (myogenic) and brown adipogenic lineages [86,111].

*MiR-129:* MiR-129 is a positive regulator of BAT function which is involved in thermogenesis and energy expenditure [92,112]. This miRNA targets both *Igf2* (insulin like growth factor 2) and *Egr1* (Early growth factor response 1), with both these factors being UCP1 inhibitors [92,112]. A recent study by Fu et al. showed that MiR-129 also directly targets *ATG7* (Autophagy-related gene 7), which is an essential autophagy gene [89]. Indeed, this study showed that MiR-129-5p inhibits adipogenesis by autophagy, demonstrating increased expression of MiR-129-5p in the AT of *db*/*db* mice. Furthermore, MiR-129-5p inhibition was shown to significantly inhibit adipocyte differentiation and white adipocyte browning in vitro, decreasing the level of specific markers (e.g., FABP4, UCP1, and PPARG) in mature white and brown adipocytes [89]. Elevated serum MiR-129-5p levels were also documented in patients with obesity, suggesting that this miRNA maybe a potential obesity-related biomarker [89].

## 3. MicroRNAs Regulating BAT and Brite

Compelling evidence indicates that an increasing number of miRNAs appear to regulate both BAT and brite AT (Figure 2), including MiRNA-455, MiRNA-30b/c, MiRNA-34a, MiRNA-27, MiRNA-378, MiRNA-133, MiRNA-155, and MiRNA-32.

*MiR-455:* MiR-455 is a key positive regulator of brown/beige AT [90,91], exhibiting BAT specific expression which can be induced upon cold exposure or bone morphogenetic protein-7 (BMP-7) stimulation [90]. Zhang and colleagues have elegantly demonstrated that MiR-455 targets *Runx1t1*, Necdin, and hypoxia inducible factor 1 subunit alpha inhibitor (*Hif1an*), reducing the hydroxylation of Asn173 on AMP-activated protein kinase alpha1 (AMPKa1) by *Hif1an*, and thus enhancing AMPKa1 activity [90]. This leads to increased Pgc1a expression, mitochondrial biogenesis, as well as the expression of genes involved in fatty acid mobilization and lipolysis [90]. Furthermore, this study also demonstrated that MiR-455 overexpression in committed brown and white pre-adipocytes and non-committed multipotent progenitors promotes cell differentiation, increasing lipid accumulation and expression of adipogenic and brown adipocyte genes (*Ucp1*, *Pparg* and *Pgc1a*) [90]. Adipose-specific MiR-455 transgenic mice were reported to show marked browning in inguinal WAT and scWAT, higher maximal thermogenic capacity, improved cold resistance, insulin sensitivity, glucose tolerance, and reduced weight gain upon HFD, as well as better lipidemic profiles [90]. Moreover, MiRNA-455 knockdown mice have been shown to have decreased BAT and WAT mass and suppressed expression of *Ucp1*, *Pgc1a, Pparg* in BAT, and *C/ebpa* in scWAT [90]. MiR-455 inhibition was also shown to suppress brown adipogenesis and reduce the expression of *Ucp1* and *Pgc1a* in brown adipocytes [90].

Another study by Cai et al. demonstrated that *Ucp1* is a target for MiR-455 during adipogenic differentiation in 3T3-L1 pre-adipocytes [91]. Indeed, this study showed that MiR-455 downregulates *Ucp1* expression through interaction with a target site of MiR-455 in the coding region of mouse *Ucp1* [91]. Another study by Pahlwani et al. showed MiR-455 thermogenic effects by targeting key brown adipogenic signaling molecules including *Hif1an*, *Pparg*, and type III transforming growth factor-β receptor (*Tgfbr3*) [92]. Notably, *Hif1an* appears to serve as an intermediate target between MiR-455 and Notch1, with MiR-455 reducing Notch1 expression which, in turn, promotes WAT browning [92]. In addition, in this study, MiR-455 also downregulated *Tgfbr3* and *Smad2*/*3* (a member of its downstream pathway), leading to enhanced energy expenditure and improved metabolic profile [92].

*MiR-30b/c:* The miRNAs of the MiR-30 family are highly expressed in BAT and WAT, and are known to promote adipogenesis and inhibit osteogenesis [93,94]. MiR-30a regulates WAT as will be discussed in the following section [113], whilst MiR30b/c are the main positive regulators of brown and brite adipogenesis [93]. MiR-30b/c levels are markedly increased during adipocyte differentiation and are stimulated by cold exposure or β-adrenergic receptor activation [93]. MiR30b/c directly targets *Rip140*, a corepressor of the thermogenic genes, and promotes thermogenesis and development of brite AT [93,94]. As such, MiR30b/c overexpression induces mitochondrial and thermogenic genes (*Ucp1*, *Cidea*) and mitochondrial respiration in brown and brite AT [93]. Moreover, MiR30b/c also enhances the expression of thermogenic genes in SVF derived from inguinal WAT [93], whist MiR30b/c overexpression also induces thermogenic genes in subcutaneous WAT (scWAT) [93,94].

*MiR-34a*: MiR-34a is a negative regulator of brown and brite adipogenesis, exhibiting increased expression in obesity [114]. This miRNA directly targets fibroblast growth factor receptor 1 (*Fgfr1*) and disrupts FGF21 signaling in AT, thus preventing PGC1A activation and browning of WAT [114]. As such, MiR-34a downregulation promotes FGF21 signaling which induces increased nicotinamide adenine dinucleotide (NAD+) levels, *Sirt1* expression and PGC1A deacetylation and browning that improves the overall metabolic profile [114]. Accordingly, MiR-34 blockage leads to reduced adiposity, improved circulating levels of insulin, FGF21, glucose, triglycerides and FFA, as well as increased mitochondrial DNA copy number and oxidative function in WAT [71,110]. The latter was also shown by MiR-34a downregulation in differentiated 3T3-L1 adipocytes [114].

Moreover, MiR-34a downregulation has been shown to act against HFD-induced obesity in mice, reducing adiposity and leading to normalization of food intake, decreased body weight and WAT mass [114]. MiR-34a inhibition also promotes brown and beige adipogenesis by regulating brown adipogenic markers (UCP1, PRMD16, PGC1α) and enhancing mitochondrial function in BAT and WAT [114]. Interestingly, Ge et al. have reported that myostatin post-transcriptionally inhibits fibronectin type III domain containing 5 (*Fndc5*) expression via MiR-34a in both myoblasts and adipocytes [95]. Indeed, myostatin appears to regulate *Fndc5*/Irisin expression and secretion (irisin represents a cleaved and secreted fragment of the FNDC5 membrane protein) via a MiR-34a-dependent mechanism, with loss of myostatin in mice leading to increased *Fndc5*/Irisin expression which contributes to WAT browning [95]. In this context, it also noteworthy that irisin has been reported to induce WAT browning via activation of *Ucp1* expression [115]. These latter effects remain to be further confirmed by additional research studies.

*MiR-27b*: MiR-27b is an inhibitor of brown and beige adipogenesis, exhibiting decreased expression in response to cold exposure and β-adrenergic activation [97]. MiR-27b targets *Prmd16*, *Ppara*, *Pparg*, cAMP-response element binding protein (*Creb*), *Pgc1b* and prohibitin, and is downregulated during WAT and brite differentiation [96,97,98]. MiR-27b is the upstream inhibitor of *Pparg* in WAT differentiation, and its inhibition leads to enhanced expression of brown adipogenic marker genes (*Ucp1*, *Prmd16*, *Ppara/g*, *Cidea*, *Pgc1a*) in pre-adipocytes from inguinal WAT and visceral AT, as well as an increased number of brite cells in inguinal WAT [116]. Furthermore, Yu et al. have shown that high expression of MiR-27b-3p in epididymal WAT inhibits browning and leads to visceral AT accumulation, with MiR-27b-3p inhibition enhancing browning in epididymal WAT of HFD-fed mice and leading to weight loss and improved insulin sensitivity [116].

*MiR-378:* MiR-378 is expressed within the *PGC1B* gene and is considered a positive regulator of BAT and a negative regulator of brite adipogenesis [117]. Indeed, Pan et al. have shown that phosphodiesterase 1b—PDE1B is a phosphodiesterase that catalyzes the cGMP and cAMP turnover [118]—is targeted by MiR-378 in BAT, but not in WAT [117,118]. This leads to diminished degradation of cAMP to AMP, with elevated cAMP levels activating the PKA and downstream signaling pathways [117]. Overall, MiR-378 has been shown to promote BAT mass, BAT oxygen consumption and *Ucp1* expression, showing increased expression during brown adipocyte differentiation [117]. As such, MiR-378 has been shown to protect against both genetic and HFD-induced obesity in mice [117]. Moreover, MiR-378 transgenic mice exhibit increased BAT mass and cell number, reduced WAT mass and increased energy expenditure [117]. Interestingly, this study also noted that the expansion of BAT, rather than the MiR-378 per se, resulted in suppressed formation of beige adipocytes in scWAT [117]. Finally, decreased *Ucp1* and *Prmd16* expression in inguinal WAT was shown with overexpression of MiR-378, without changes in the levels of the adipogenic markers PPARG and AP2 [117]. Notably, Kim et al. have shown that eicosapentaenoic acid (an omega 3 fatty acid) binds to and activates the free fatty acid receptor 4 (FFAR4; a functional receptor for polyunsaturated fatty acids), which positively modulates MiR-378, resulting in increased *Ucp1* expression and cAMP levels [99].

In addition, Zhang et al. reported that MiR-378 prevents and treats obesity in mice by activating the pyruvate-phosphoenolpyruvate futile cycle in the muscle and enhancing lipolysis in adipose tissues [119]. This study also highlighted that transgenic mice overexpressing MiR-378 provide a genetic system for investigating the metabolic crosstalk between different tissues at the whole-body level [119]. Overall, MiR-378 appears to mediate a wide range of biological processes implicated in cancer, angiogenesis, hepatosteatosis, and T2DM [120].

*MiR-133*: MiR-133 has two isomers (MiR-133a and -133b) with highly similar sequences, differing only at the 3′-terminal base, with MiR-133a-1/2 and MiR-133b terminating with G-3′ and A-3′, respectively [121]. MiR-133 is a negative regulator of brown adipogenesis, acting as an inhibitor of BAT differentiation which targets *Prmd16* [100,101,102]. MiR-133 is highly expressed in BAT and inguinal WAT, whilst it is also enriched in muscle tissue and myogenic cells [100]. Downregulated MiR-133 levels in BAT and scAT are noted after cold exposure as a result of decreased expression of its transcription regulator myocyte enhancer factor-2 (*Mef2*), resulting in increased *Prmd16* expression and upregulation of thermogenic genes [100]. Double knockout of MiR-133a (there are two alleles for MiR-133a with identical sequences, namely, MiR-133a1 and MiR-133a2, which are located on different chromosomes) in mice increased the program of thermogenic genes in scAT and inguinal WAT, resulting in browning/beigeing of these cells [100].

Furthermore, Yin et al. showed that MiR-133 overexpression in primary brown pre-adipocytes strongly impaired their adipogenic differentiation, and decreased the levels of browning genes (*Ucp1*, *Cidea*, *Pgc1a*, *Prmd16*) and general adipogenic genes (*Adipoq*, *C/ebpb*, *Fabp4* and *Pparg*) [101]. Taken together, these studies indicate that MiR-133 (MiR-133a and -133b) plays a critical role in suppressing brown adipogenesis via inhibition of *Prmd16*.

*MiR-155:* MiR-155 is another negative regulator of brown adipogenesis which targets *C/ebpb* [103,104,105]. MiR-155 is enriched in BAT, being highly expressed in proliferating brown pre-adipocytes and exhibiting declining expression with cell differentiation [103,104,105]. Of note, C/EBPB is crucial for brown and beige adipocyte differentiation, with *C/ebpb* knockdown resulting in BAT defects [122]. MiR-155 is negatively regulated by C/EBPB, thus forming a double feedback loop [103]. Moreover, MiR-155 is positively regulated by transforming growth factor beta (TGFB), exhibiting induction upon TGFB treatment [103,105]. MiR-155 deficient mice show a high cellular respiration rate and increased number of brown-like adipocytes in inguinal WAT after cold exposure [103]. Similarly, HFD-fed MiR-155 knockout mice were shown to gain 56% less weight in comparison to wild type [104]. MiR-155 deficient adipocytes showed upregulated brown (*Ucp1*, *Cidea*, *Pparg*) and white (*Fabp4*; *Adipoq*; fatty acid synthase; and Patatin-like phospholipase domain-containing protein 2, *Pnpla2*) adipogenic genes and glucose metabolism genes (Insulin receptor substrate 1, *Irs1*; and Glucose Transporter Type 4, *Glut4*) [104]. Overall, MiR-155 deletion can increase thermogenesis and insulin sensitivity, whilst limiting inflammation in WAT and preventing diet-induced obesity in mice [104]. Indeed, transgenic mice overexpressing MiR-155 show reduced BAT mass, altered BAT morphology and lower levels of thermogenic markers without alteration in body weight and food intake [103].

Similarly, Karkeni et al. showed that overexpression of MiR-155 is associated with increased inflammatory state in adipocytes [105]. This study showed up-regulation of MiR-155 in biopsies of subjects with obesity where the induction of MiR-155 was correlated with tumor necrosis factor alpha (*TNFA*) expression and BMI [105].

*MiR-32:* MiR-32 is a positive regulator of brown adipogenesis with significantly increased expression in BAT during cold exposure, promoting BAT thermogenesis and inguinal WAT browning [106]. As such, MiR-32 has been shown to be a BAT specific super enhancer which targets *Tob1* (Transducer of erbB2 1; a p38/AMPK signaling repressor) [106]. Targeting *Tob1* or diminishing its repressive effect on p38/MAPK results in ATF2 (Activating Transcription Factor 2) phosphorylation and activation of enhanced BAT thermogenesis and transactivation of WAT browning [106]. Interestingly, BAT-specific MiR-32 overexpression has been shown to result in increased BAT thermogenesis, as well as increased FGF21 serum levels which further promote WAT browning [106]. Therefore, MiR-32 and *Tob1* appear to be modulators of FGF21 signaling, which can be manipulated for therapeutic effects against obesity and the metabolic syndrome [106].

## 4. Micro RNAs Regulating Brite AT

MiR-196a, MiR-26, MiR-125, and Let-7 are key miRNAs, which have been reported to mainly regulate brite AT (Table 2 and Figure 2).

*MiR-196a:* MiR-196a targets certain homeobox (*HOX*) genes in human pre-adipocytes [127], playing a role in the browning of white progenitor cells and inducing WAT browning with enhanced expression of brown adipogenic genes (*C/ebpb*, *Prmd16*, *Ucp1* and *Pgc1a*) [78]. Indeed, MiR-196a is a positive regulator of brite adipogenesis and promotes browning by directly binding to and suppressing Homeobox C8 (*Hoxc8*, a determinant of white adipogenesis) [78]. MiR-196a expression is induced by cold exposure and β-adrenergic stimulation, whilst HOXC8 directly suppresses the expression of *C/ebpb* in cooperation with histone deacetylase 3 (HDAC3) in scAT [78]. Notably, mice overexpressing MiR-196a showed resistance to obesity and improved glucose metabolism [78]. Furthermore, Divoux et al. demonstrated higher MiR-196a expression in the gluteal AT depot leading to decreased fat development in this depot, whilst higher MiR-196a expression in the abdominal depot seems beneficial and associated with decreased fat mass [127]. Finally, using functional studies and transcriptomic profiling of MiR-196a knockdown pre-adipocytes, Hilton et al. showed that MiR-196a regulates pre-adipocyte proliferation and extracellular matrix pathways, highlighting that this miRNA is involved in the regulation of human body fat distribution [128]. This study also documented that a single nucleotide polymorphism (rs11614913) in pre-MiR-196a-2 influences the expression of MiR-196a in abdominal scAT and is associated with the waist-to-hip ratio [128].

*MiR-26*: MiR-26a and MiR-26b are identified as key regulators of human white and beige adipocyte differentiation, constituting positive regulators of brown adipogenesis that targets ADAM metallopeptidase domain 17 (ADAM17) [123], which is an anti-adipogenic and anti-browning factor [129]. The MiR-26 family consists of three members (MiR-26a1, MiR-26a2 and MiR-26b), which are upregulated in early adipogenesis, and their inhibition prevents lipid accumulation, whilst their overexpression accelerates it [124]. MiR-26 levels increase during WAT differentiation, and MiR-26a is enriched in BAT and induced in WAT upon cold response [123]. MiR-26a promotes *UCP1* and *PGC1A* expression and can induce oxygen consumption in human brite adipocytes [123]. Moreover, mimics of MiR-26a/b have been shown to promote white and beige adipogenesis causing induction of brown adipogenic markers (UCP1, PGC1A, AP2, and PRDM16) and brown-like mitochondrial morphology [123].

Of note, a recent study by Acharya and colleagues reported that MiR-26 plays a critical role in regulating adipogenesis in vitro and in vivo by suppressing adipocyte progenitor cell differentiation and fat production by targeting *Fbxl19* (gene encoding the F-box and leucine rich repeat protein 19; FBXL19), which encodes a component of Skp–Cullin–F-box-containing (SCF) E3 ubiquitin ligase complexes [124]. Indeed, FBXL19 mediates targeted ubiquitylation of proteins destined for proteasomal degradation [130], and appears to be a novel MiR-26 target that functions downstream from this miRNA to drive adipogenesis [124]. Interestingly, loss of MiR-26 results in dramatic AT expansion early in adult life and triggers precocious adipocyte progenitor cell differentiation, resulting in adipocyte hyperplasia and WAT expansion in a manner that resembles the physiologic response to caloric excess [124]. Over time, excessive adipocyte progenitor cell differentiation leads to depletion of this progenitor compartment, as seen in older MiR-26-triple-knockout mice (knockout of MiR-26a-1, MiR-26a-2, and MiR-26b) which exhibit impaired weight gain and adipogenesis when fed a HFD [124]. However, MiR-26 overexpression in the adipocyte progenitor cells lineage was sufficient to block HFD-induced weight gain, adipose tissue expansion, hyperglycaemia, and hyperlipidaemia [124].

*MiR-125:* The MiR-125 family consist of two subfamilies: MiR-125a and MiR-125b, each further divided into 3p and 5p, which respectively derive from the 3′- or 5′-end of pre-MiR-125a and pre-MiR-125b [131]. MiR-125b-5p is identified as negative regulator of browning and the formation of functional brite adipocytes [132]. MiR-125b-5p is highly expressed in WAT and low in BAT; however, its expression decreases during browning and shows a proportional inverse association to UCP1 expression [132]. Notably, injection of MiR-125b-5p into inguinal WAT can inhibit brite adipocyte formation and mitochondrial biogenesis [132]. MiR-125b-5p overexpression in scAT of mice attenuates the *Ucp1*, *Cidea*, and *Prmd16* expression and reduces large lipid droplets [132]. Furthermore, inhibition of this miRNA increases UCP1 and CIDEA levels and results in smaller lipid droplets [132]. Similarly, overexpression of MiR-125b-5p in human multipotent adipose-derived stem (hMADS) cells inhibited oxygen consumption and mitochondrial DNA content [132]. Using microarray-analysis, Rockstroh and colleagues showed that MiR-125b-5p is upregulated during human adipocyte differentiation and identified the matrix metalloproteinase 11 (MMP11) as a direct target of MiR-125b-5p [125]. MMP11 overexpression decreased AT accumulation, indicating that MMP11 acts as an anti-adipogenic regulator, whereas overexpression of MiR-125b-5p itself reduced adipogenesis [125].

*Let-7*: The Let-7 family of miRNAs is composed of 13 miRNA precursors giving rise to 10 distinct mature Let-7 miRNAs, with highly conserved sequence and function across species [70]. Dysregulated expression of the Let-7 family is associated with various diseases, such as cancer, lung, and CVD [126]. Furthermore, the Let-7 family is reported to target high-mobility group AT-hook 2 *(HMGA2)* and is considered involved in adipogenesis, glucose metabolism, insulin resistance, and inflammation [133,134]. A recent study by Youssef et al. showed that Let-7 is an important regulator of adipogenesis in 3T3-L1 cells, with Let-7a-5p being downregulated in HFD-fed rats [135]. Let-7 is upregulated after induction of adipogenesis by either the standard adipogenic cocktail containing dexamethasone, isobutylmethylxanthine, and insulin or the combination of rosiglitazone and insulin [133]. Furthermore, Let-7 is much more abundant in mature adipocytes than pre-adipocytes derived from mouse epididymal AT [133]. Let-7 overexpression has been shown to block 3T3-L1 cell growth and completely inhibit terminal differentiation as measured by lipid accumulation and marker genes [133].

Finally, Let-7i-5p is also a thermogenesis inhibitor, strongly inhibiting mitochondrial and browning marker genes (e.g., *Ucp1*, *Prmd16*, *Cidea*, and citrate synthase) in mice scAT and human and murine brite adipocytes [126]. Injection of Let-7i-5p into murine scAT impaired the formation and function of brite adipocytes, whilst significant reduction in Let-7i-5p expression was noted upon β3-adrenergic stimulation [126]. Let-7i-5p overexpression led to inhibition of basal oxygen consumption and decreased *UCP1* expression in hMADS brite adipocytes [126]. However, using miRNA-target prediction tools, this study showed that *UCP1* was not a putative target of Let-7i-5p [126]. Overall, Let-7i-5p levels are associated with brite adipocyte formation and function in both mice and humans and may represent a potential new regulator of energy expenditure [126]. Additional targets for Let-7 have also been identified, including *E2f6* (E2F transcription factor 6), *Cdc34* (cell division cycle 34), and *Igf2bp1* (insulin-like growth factor 2 mRNA-binding protein 1) genes [136,137,138,139].

## 5. MicroRNA in WAT

An array of miRNAs have been identified in WAT, which are largely involved in the regulation of adipocyte differentiation and function (Table 3 and Figure 2) [140]. Due to its energy storage role, WAT undergoes dynamic changes which reflect the balance between energy intake and expenditure, with excess energy intake resulting in increased lipid storage and differentiation of pre-adipocytes to mature adipocytes [141]. Increasing data show that this differentiation of pre-adipocytes is further characterized by changes in the miRNA expression profile of WAT, including both upregulated miRNAs (e.g., MiR-103, MiR-107, MiR-143, MiR-26b, MiR-375, MiR-21, MiR-148a, and MiR-30) [94,142,143,144,145,146,147] and downregulated miRNAs (e.g., MiR-221, MiR-155, MiR-424, MiR-210, and MiR-125b) [143,148,149]. As WAT and BAT adipogenesis share common pathways, there is an overlap in the miRNAs identified in both WAT and BAT, which mediate various effects [51,135].

*MiR-181:* The MiR-181 family is composed of six mature miRNAs (MiR-181a-1, MiR-181a-2, MiR-181b-1, MiR-181b-2, MiR-181c, and MiR-181d) that are highly expressed in multiple tissues and are encoded in three independent paralog precursor transcripts on three corresponding chromosomes [175]. The MiR-181 family has been identified as a non-redundant metabolic rheostat/regulator, regulating the phosphatidylinositol 3-kinase (PI3K)/phosphatase and tensin homolog deleted on chromosome 10 (PTEN) signaling axis [151]. Of note, MiR-181a targets *TNFA* and promotes adipogenesis [150].

Interestingly, gut microbiota appear to impact on the expression of the MiR-181 family in white adipocytes in mice and, thus, is further implicated in the regulation of key pathways controlling adiposity, insulin sensitivity, and WAT inflammation [152]. Indeed, a HFD is shown to result in altered composition of the gut microbiome, with aberrant MiR-181 overexpression in WAT adipocytes and development of obesity, insulin resistance, and WAT inflammation [152]. Moreover, Virtue et al. showed that the MiR-181 family represses several critical genes in pathways controlling insulin signaling and WAT adipogenesis [152]. As such, this regulation of MiR-181 may serve as a rheostatic mechanism which allows rapid and global modulation of gene expression programs in white adipocytes in response to microbiota-derived signals [152]. Overall, these findings suggest that MiR-181 regulation in WAT may constitute an additional potential mechanism by which the gut microbiota can control mammalian metabolism. Thus, the MiR-181 family may represent a potential novel therapeutic target to modulate WAT function in the context of obesity.

*MiR-30a*: *MiR-30a* is shown to directly target the signal transducer and activator of transcription 1 (*STAT1*) in both human and mouse cells, with MiR-30a expression in scAT protecting adipocytes from detrimental effects of pro-inflammatory cytokines and leading to improved insulin sensitivity in obese mice [113]. As such, MiR-30a expression in adipocytes may have an anti-inflammatory effect mediated through direct suppression of the STAT1 signaling pathway [113]. However, STAT1 activation inhibits MiR-30a expression in adipocytes, potentially contributing to the chronic inflammatory state that characterizes obesity [113]. Moreover, the MiR-30 family is highly conserved [176,177,178] and may play a role in modulating the polarization of macrophages in visceral AT through regulation of Delta-like protein 4 (DLL4; a Notch1 ligand that contributes to metabolic disease and macrophage inflammation), with downregulation of several miRNAs of the MiR-30 family (MiR-30a-5p, MiR-30c-5p, and MiR-30e-5p) in adipose tissue macrophages in obesity [153]. Indeed, Miranda et al. showed upregulation of *DLL4* in adipose tissue macrophages in obesity, whilst MiR-30 inhibition in vitro triggered DLL4-Notch1 signaling and pro-inflammatory responses in macrophages [153].

Furthermore, overexpression of MiR-30a-5p also decreased M1 polarization in the RAW264.7 macrophage cell line (M1 and M2 macrophage polarization contribute to pro-inflammatory and anti-inflammatory responses, respectively), suggesting that MiR-30 is involved in attenuating M1 macrophage activation through regulation of the DLL4-Notch signaling pathway [153]. Interestingly, a HFD appears to contribute to DNA hyper-methylation of MiR-30 genes, leading to MiR-30 downregulation and promoting inflammation and insulin resistance in obesity via increased DLL4-Notch signaling [153]. Thus, this DNA methylation may attenuate MiR-30 expression, suggesting important roles for miRNAs and DNA methylation in the regulation of adipose tissue macrophage polarization and obesity-induced insulin resistance [153]. Finally, Youssef et al. have shown that MiR-30a-5p targets genes implicated in AMPK signaling, with a HFD resulting in downregulation of this miRNA [135].

*MiR-143:* MiR-143 has been identified as an important regulator of adipocyte differentiation in both humans and mice, targeting the extracellular-signal-regulated kinase 5 (*ERK5*) [155,179,180]. ERKs inhibit adipogenesis in the later stages of differentiation by phosphorylating PPARG, thus MiR-143 promotes adipocyte differentiation via the ERK pathway, specifically via ERK5 [181]. MiR-143 inhibition suppresses adipogenic marker genes (hormone-sensitive lipase, *GLUT4, AP2, PPARG*) and triglyceride accumulation in scAT pre-adipocytes [155]. In human pre-adipocytes, Esau et al. showed that MiR-143 is induced during differentiation, while MiR-143 inhibition with antisense oligonucleotides blocked this differentiation [155]. Similarly, Chen et al. reported that MiR-143 enhances the differentiation of cultured human pre-adipocytes and directly targets *FGF7,* which may function as a fine-tuning molecule in adipogenesis [154].

In addition, bioinformatic analyses have predicted that MiR-143 can target the mitogen-activated protein kinase 7 (*MAP3K7*) gene, which is involved in the TGFB pathway [158]. TGFB acts via SMAD proteins (SMAD2 and SMAD3) to inhibit the induction of transcription factors that regulate adipogenesis (e.g., PPARG, C/EBPA and ADD1) [182,183]. As such, TGFB inhibits adipocyte differentiation, with decreased surface expression of TGFB receptors during adipocyte differentiation [182]. Therefore, although the exact effects of MiR-143 on this pathway are not clarified yet, MiR-143 appears to target the TGFB pathway by reducing cell surface expression of TGFB receptors or inhibiting SMAD proteins [158]. Furthermore, FFA and leptin can significantly reduce the expression of MiR-143 in human visceral adipocytes [184]. Since leptin has been previously found to inhibit adipogenesis [185], the findings by Zhu et al. suggest that this can be through MiR-143 downregulation [184]. Indeed, in obesity, there is an increase in FFA and leptin release by WAT adipocytes that can act in an autocrine manner to further cause adipocyte dysfunction [14].

Of note, MiR-143 may also induce insulin resistance in vascular smooth muscle cells exposed to adipocyte-derived factors and in hepatocytes from MiR-143 transgenic mice by downregulating the expression of oxysterol-binding protein-related protein 8 (OPR8), which impairs protein-kinase B (AKT) activation and ultimately downstream insulin signaling [156,186]. Moreover, compared to controls, significantly elevated MiR-143-3p levels in serum and urine have been found in patients with metabolic syndrome, with the insulin-like growth factor 2 receptor (*IGF2R*) gene being among the target genes of MiR-143-3p [157]. As such, MiR-143-3p knockdown was shown to protect against insulin resistance in patients with metabolic syndrome via targeting of *IGF2R* and activation of the insulin signaling pathway [157]. However, MiR-143-3p overexpression was shown to impair insulin-stimulated AKT activation and glucose uptake in 3T3-L1 pre-adipocytes [157].

*MiR-103* and *MiR-107:* MiR-103 and MiR-107 have similar nucleotide sequences, with the exception of one nucleotide at the 3′ end [187]. Increased MiR-103 expression has been reported during adipocyte maturation, with ectopic expression of this miRNA accelerating the expression of pro-adipogenic genes and lipid accumulation, whereas antisense inhibition of MiR-103 reverses these effects [143,159,188,189]. Whilst investigating the effects of MiR-103 on porcine pre-adipocyte differentiation, Li et al. found that MiR-103 inhibition resulted in increased mRNA and protein expression of retinoic acid-induced protein 14 (RAI14) in the early stages of adipogenesis [159]. RAI14 is abundantly expressed in porcine pre-adipocytes, with its expression declining during differentiation [159]. Therefore, MiR-103 may target the *RAI14* gene and reduce its expression, to promote adipogenesis [159]. Interestingly, retinoic acid (a metabolite of vitamin A) is suggested to exert anti-adipogenic effects potentially through *RAI14* induction [159,190]. Furthermore, MiR-103 can promote adipogenesis through activation of the protein kinase B/mammalian target of rapamycin signaling pathway (AKT/mTOR pathway; with an important role in promoting adipogenesis) [191,192,193], resulting in enhanced *Pparg* expression in murine pre-adipocytes [189]. Indeed, inhibition of MiR-103 has been shown to abolish these effects [189]. Finally, MiR-103 reverses the anti-adipogenic effects of myocyte enhancer factor 2D (MEFD2), which is a transcription factor that negatively regulates pre-adipocyte differentiation by downregulating the expression of multiple adipocyte markers (PPARG, C/EBPA, FABP4, and FA synthase) [189].

Unlike MiR-103, MiR-107 has been found to suppress pre-adipocyte differentiation by targeting the cell division protein kinase 6 (*CDK6*) gene [83]. Attenuation of CDK6 signaling can directly reduce the expression of pro-adipogenic genes and cause unnecessary cell cycle arrest at the G1 phase [83]. Although MiR-103 and MiR-107 have opposing effects on adipogenesis, both of these miRNAs are increased in the liver of obese mice and are associated with endogenous glucose production and insulin resistance [160]. Notably, caveolin-1 (Cav-1; a principle protein of caveolae in adipocytes and an important regulator of insulin signaling) is downregulated by MiR-103 and MiR-107 in WAT and liver tissue [160]. Silencing of these MiRNAs results in upregulation of Cav-1 expression in both tissues, as well as decreased adipocyte size and improved insulin signaling and glucose uptake [160]. In T2DM, increased levels of MiR-103 and MiR-107 have also been found in the circulation, suggesting that these miRNAs can be potential T2DM biomarkers [194]. Finally, a recent study has also found that expression of MiR-103 and MiR-107 is increased during apoptosis of murine pre-adipocytes [161]. Moreover, it appears that MiR-103 and MiR-107 can promote endoplasmic reticulum (ER) stress-mediated apoptosis by targeting the Wnt3a/β-catenin/activating transcription factor 6 (ATF6) signaling pathway in pre-adipocytes [161]. As such, MiR-103 and MiR-107 could also be implicated in the increased adipocyte apoptosis in WAT that is noted in obesity [195].

*MiR-221*: The expression of MiR-221 is downregulated during pre-adipocyte differentiation, whilst it is upregulated in the WAT of patients with obesity [143,148,149]. Indeed, MiR-221 negatively regulates adipogenesis by attenuating the induction of *PPARG* and *C/EBPA* [196]. In obesity, dysfunctional adipocytes markedly increase the release of TNFA and leptin [197,198]. Intriguingly, Meerson et al. found that MiR-221 expression is downregulated in response to TNFA and leptin in human pre-adipocytes, mature adipocytes and neuroblastoma-derived cells [162]. As such, the mechanism by which MiR-221 is upregulated in obesity is yet to be elucidated. Moreover, MiR-221 can reduce the mRNA and protein expression of the proto-oncogene E26 transformation–specific-1 (ETS1) in human pre-adipocytes [162]. This finding is in line with a previous study which showed that MiR-221 reduces ETS1 protein expression in endothelial cells [199]. However, TNFA and leptin increase *ETS1* expression in pre-adipocytes and mature adipocytes [162], whilst ETS1 also regulates T-helper 1 (Th1) cell inflammatory responses, angiotensinogen II-mediated endothelial inflammation and vascular remodeling [200,201]. Thus, it has been suggested that TNFA and leptin exacerbate inflammation within WAT by reducing MiR-221 expression which consequently increases *ETS1* expression. However, Peng et al. have reported increased MiR-221 expression after TNFA treatment in murine pre-adipocytes and that MiR-221 inhibits Sirtuin-1 (SIRT1) protein production, resulting in increased inflammation and insulin resistance [202]. SIRT1 protects against inflammation and TNFA induced insulin resistance, whilst also promotes glucose uptake and insulin signaling in murine adipocytes [203]. Thus, an additional mechanism is suggested by which TNFA contributes to WAT dysfunction via MiR-221.

Adiponectin receptor 1 (*AdipoR1*) is another target of MiR-221, with the latter inhibiting *AdipoR1* gene translation and attenuating its expression in pre-adipocytes, muscle and liver cells [162,163]. Adiponectin is a well-established adipokine which regulates glucose homeostasis by promoting glucose uptake and suppressing endogenous glucose production, whilst also improving insulin sensitivity [204,205,206]. These effects are induced through binding of adiponectin with its receptors (ADIPOR1 and ADIPOR2) [207]. Of note, MiR-221 expression is downregulated during the differentiation of murine myocytes with concomitant upregulation of *AdipoR1* expression [163]. However, in mouse models of obesity, MiR-221 expression is elevated in liver and skeletal muscle, coinciding with low ADIPOR1 protein expression [163]. These findings suggest that MiR-221 can potentially contribute to T2DM development by impairing glucose homeostasis and insulin signaling through downregulation of *AdipoR1* expression in metabolically active tissues.

*MiR-17:* MiR-17 is reduced in human WAT in obesity, insulin resistance, and metabolic disease and circulating MiR-17 is significantly lower in metabolic disease [208,209]. Notably, Zhang and colleagues showed that MiR-17 improved inflammation-induced insulin resistance by directly suppressing the expression of the apoptosis signal-regulating kinase 1 (*Ask1*) gene in macrophages [164]. ASK1 is a member of the MAP kinase family and is involved in the regulation of macrophage activation [210]. In addition, MiR-17 indirectly increased insulin-stimulated glucose uptake by reducing the production of pro-inflammatory cytokines [210]. Indeed, MiR-17 overexpression was shown to reduce the secretion of pro-inflammatory cytokines (interleukin-6, interleukin-1B and TNFA) in LPS-stimulated macrophages, whereas all these MiR-17-induced effects were reversed by *Ask1* overexpression [164]. Overall, MiR-17 appears implicated in inflammatory responses [211,212], with inhibition of MiR-17-5p further inhibiting the activation of macrophages in T2DM [213]. MiR-17-5p also blocks the expression of *STAT3* [165], which is a major regulatory pathway of macrophage activation, thereby reducing the suppressive function of myeloid-derived suppressor cells. These studies consistently suggest that MiR-17 may play a role in preventing macrophage-mediated AT inflammation and improve insulin resistance, providing an additional target for T2DM treatment [164].

*MiR-27a:* MiR-27a is considered a crucial miRNA in WAT [140,214], which is reported to act as a negative regulator of adipogenesis [215,216]. MiR-27a is upregulated in 3T3-L1 adipocytes and *ob/ob* mice [166,167], as well as in the circulation of patients with T2DM or obesity [217]. As such, a number of studies have identified increased MiR-27a levels in patients with obesity, compared to lean controls, with correlations noted between serum MiR-27a and adipokine levels [149,218]. Notably, MiR-27a is reported to impact on M2 cytokine expression [219]. Furthermore, the PPAR family is identified as a potential target for MiR-27a [220,221], with MiR-27a upregulation having been shown to suppress the transcription regulation and expression of *Pparg* [166,222]. Indeed, MiR-27a can bind to the 3′-UTR of *Pparg* mRNA with a high score [222]. Moreover, Yao et al. reported increased MiR-27a serum levels in HFD-fed mice, with increased fat percentage and adipocyte insulin resistance [223]. In this study, increased cytokine levels were noted with MiR-27a overexpression, whilst decreased levels were documented with MiR-27a knockdown after 4 weeks of HFD [223]. Overall, it is suggested that MiR-27a in WAT might represent an important target in macrophage activation and regulate local or systematic obesity-induced insulin resistance via PPARG/nuclear factor kappa B (NFKB) [223].

*MiR-130:* MiR-130a and MiR-130b expression levels decline during the differentiation of human and murine pre-adipocytes [169]. Loss and gain of function experiments revealed that MiR-130 directly inhibits PPARG gene and protein expression, resulting in reduced triglyceride accumulation, as well as reduced expression of adipocyte-specific genes (e.g., *FABP4*, lipoprotein lipase and adipsin) [169]. MiR-130 expression was downregulated in scAT of patients with obesity compared to controls, and, accordingly, *PPARG* mRNA expression was upregulated [169]. An inverse correlation was also found between MiR-130 and BMI, suggesting that MiR-130 expression decreases with increased adiposity [169].

As aforementioned, it is well established that obesity can result in impaired adipogenesis, which contributes to AT dysfunction [224,225,226]. This adipogenesis dysregulation can be attributed to the pro-inflammatory environment of WAT in obesity, where pro-inflammatory cytokines (e.g., TNFA) can exert anti-adipogenic effects on adipocytes through downregulation of *PPARG*, *C/EBPA* and *FABP4* expression [224,225,226]. In this context, Kim et al. further reported that the negative effects of TNFA on *PPARG* expression were mediated through MiR-130 [168]. Indeed, it has been previously shown that TNFA downregulates *PPARG* via activation of NFKB [227]. These findings were confirmed by Kim et al. who also found that activation of this pathway is responsible for TNFA-induced MiR-130 expression [168]. Of note, MiR-130b alone can also increase the accumulation of macrophages in WAT of HFD-fed mice and can polarize macrophages to express the M1 phenotype by targeting *Pparg* [228]. Therefore, MiR-130b further contributes to the overall inflammatory state of WAT in obesity.

Furthermore, MiR-130 can suppress adenomatosis polyposis coli down-regulated 1 (*Apcdd1*), encoding the APCDD1 protein which is an inhibitor of the Wnt signaling pathway [170]. APCDD1 mRNA and protein expression is upregulated during pre-adipocyte differentiation and, accordingly, expression of Wnt proteins is decreased [170]. In both subcutaneous and visceral AT, diet-induced obese mice expressed significantly higher levels of MiR-130, which corresponded to decreased APCDD1 levels and increased Wnt3a levels [170], resulting in reduced adipogenesis in these tissues. Compared to controls, reduced expression levels of APCDD1 and adipogenic markers (PPARG and C/EBPA) were also observed in scAT of patients with obesity [170]. Interestingly, both genetic and diet-induced mouse models of obesity have increased circulating MiR-130 levels and increased MiR-130 expression in WAT [168,229].

Similarly, several studies have reported increased circulating levels of MiR-130b in individuals with obesity, which positively correlated with metabolic parameters, including BMI, insulin resistance and triglyceride levels [229,230,231]. In accordance with this, Wang et al. found that TGFB, which is increased in the AT of obese mice and has well-known anti-adipogenic effects [232,233], can increase MiR-130b secretion via neutral sphingomyelinase 2 [227]. This effect can potentially explain the increased circulating MiR-130b levels observed in obesity. Similarly, Ortega and colleagues reported decreased circulating MiR-130b levels in T2DM patients with and without obesity [234] as well as decreased expression of MiR-130b in scWAT of patients with obesity (with or without coexisting T2DM) [148]. A more recent study measuring MiR-130 (a/b) serum levels in patients with coronary artery disease (CAD) with and without T2DM showed significantly decreased circulating MiR-130 levels in non-diabetic patients with CAD and even more decreased in patients with CAD and T2DM [235]. The metabolic dysregulation that is associated with T2DM may explain, at least partly, why patients with T2DM express lower circulating MiR-130 levels compared to patients with only obesity.

*MiR-33:* MiR-33a and MiR-33b, which belong to the MiR-33 family, are found in the introns of genes encoding sterol regulatory element-binding proteins (SREBPs); *SREBP2* and *SREBP1*, respectively [236]. These proteins are involved in the synthesis of fatty acids and cholesterol, as well as cholesterol uptake [236]. Although MiR-33b is upregulated during adipogenesis, this miRNA has been reported to negatively regulate porcine and human pre-adipocyte differentiation [173,237]. Overexpression of MiR-33b can attenuate lipid accumulation and downregulate adipogenic markers such as PPARG, C/EBPA, GLUT4, and adiponectin [237]. Furthermore, MiR-33b can decrease mRNA and protein expression of HMGA2 [173]. HMGA2 is a transcription factor that is upregulated in the early stages of differentiation and, together with STAT3, regulates pre-adipocyte proliferation and differentiation [238,239]. Silencing of this transcription factor has similar effects to overexpression of MiR-33, attenuating adipogenesis [173].

A well-studied target of MiR-33 is the gene encoding the adenosine triphosphate–binding cassette transporter (ABCA1), which regulates cholesterol efflux and HDL synthesis, with these processes being attenuated by MiR-33 [171,172]. Of note, genetic ablation of MiR-33 in mice (MiR-33^−/−^) leads to increased body weight, elevated levels of circulating cholesterol, impaired glucose metabolism, and hepatosteatosis [240]. In addition, MiR-33 knockdown has been shown to increase macrophage accumulation within WAT and, hence, augment inflammation [240]. The expression of key enzymes involved in lipolysis was also downregulated in WAT, contributing to impaired FFA release in response to lipolytic stimuli (e.g., insulin) [241]. Moreover, *Hmga2* expression was significantly higher in MiR-33^−/−^ mice, thus promoting WAT expansion and increased cellular lipid accumulation [241]. However, pharmacological inhibition of MiR-33 is being considered for preventing atherosclerosis, with MiR-33 inhibitors promoting fatty acid oxidation and improving the lipidemic profile (decreased triglycerides and increased HDL levels) [172]. Contrary to genetically silencing MiR-33, MiR-33 inhibition can promote M2 macrophage polarization in AT [242]. Notably, Karunakaran et al. further reported that MiR-33 inhibitors did not affect any other metabolic factors that were associated with HFD in mice, suggesting that this pharmacological inhibition may be effective and safe in treating atherosclerosis [242].

*MiR-369-5p:* MiR-369-5p appears to negatively regulate adipogenesis [174], directly targeting *FABP4* (FABP4 is responsible for the transportation of fatty acids to different parts of the cell and their storage as lipid droplets) [243]. Ectopic expression of MiR-369-5p results in downregulation of the *FABP4* gene expression without affecting the expression of PPARG, which is a regulator of FABP4 [174,243]. To date, the role of MiR-396-5p in regulating WAT has not been extensively studied and, thus, further research is required to clarify its exact role in WAT.

## 6. Conclusions

Obesity is a worldwide epidemic, posing a significant disease burden with a still unmet need for novel effective therapies. A number of miRNAs have been identified to play key regulatory roles in BAT, brite AT and WAT by acting on transcription factors to promote or inhibit adipocyte differentiation, thus regulating key signaling pathways of adipogenesis. Our understanding of such miRNA-induced effects in AT is rapidly growing, in parallel to advances in AT biology and the recent heightened attention on the role of both BAT and browning of WAT in humans. In this context, an increasing number of AT-related miRNAs have been identified as biomarkers and potential therapeutic targets for obesity and obesity-related cardio-metabolic diseases. For example, since MiR-103 and MiR-107 have been shown to regulate insulin resistance and that antagomiR-mediated MiR-103/107 silencing significantly improves insulin sensitivity and glucose tolerance in obese mice [160], anti-miR-103/107 oligonucleotides (anti-miRs) are explored for the treatment of T2DM and NAFLD (e.g., phase I/IIA randomized clinical trial of antagomiR-103 and -107 in subjects with T2DM and NAFLD; ClinicalTrials.gov: NCT02826525). However, the clinical applicability of such novel miRNA-based therapies requires additional trials, which will address their efficacy, as well as potential interactions and safety concerns [244,245]. Overall, it is evident that further rigorous research is required in this field to exploit the full potential of the burgeoning diagnostic and therapeutic implications of these novel regulators of AT differentiation and function.

## Figures and Tables

**Table 1:** Selected key microRNAs (miRNAs) and their reported effects in brown adipose tissue (BAT).

miRNA	Function(s)	Target(s) *	Reference(s) and Corresponding Study Model(s)
MiR-193b-365	Involved in the regulation of BAT differentiation	*Runx1t1*	[85]: Mouse; in vitro [86]: Mouse; in vitro
MiR-182	Positive regulator of BAT adipogenesis	*Insig1, Pdgfr2*	[77]: Mouse; in vitro
MiR-203	Positive regulator of BAT adipogenesis	*Insig1, Pdgfr2*	[77]: Mouse; in vitro
MiR-106b-93	Negative regulator of BAT differentiation, involved in energy homeostasis	*Ppara*	[87]: Mouse
MiR-328	Positive regulator of BAT differentiation, controls brown adipogenesis by regulating the switch between muscle-specific (myogenic) and brown adipogenic lineages	*Bace1*	[86]: Mouse; in vitro [88]: Mouse; in vitro
MiR-129	Positive regulator of BAT function, involved in thermogenesis and energy expenditure, potential obesity biomarker	*Igf2*, *Egr1*	[89]: Human; mouse; in vitro
MiR-455	Positive regulator of brown/beige AT, promotes cells differentiation	*UCP1, Runx1t1,* Necdin, *Hif1an, Tgfbr3*	[90]: Human; mouse; in vitro [91]: In vitro [92]: Mouse
MiR-30b/c	Positive regulator of brown and brite adipogenesis	*Rip140*	[93]: Mouse; in vitro [94]: In vitro
MiR-34a	Negative regulator of brown and brite adipogenesis, exhibiting increased expression in obesity	*Fgfr1*	[95]: Mouse; in vitro
MiR-27b	Inhibitor of brown and beige adipogenesis, exhibiting decreased expression in response to cold exposure and β-adrenergic activation	*Prdm16, Ppar, Pparg, Creb, Pgc1b,* Prohibitin	[96]: In vitro [97]: Mouse; in vitro [98]: In vitro
MiR-378	Positive regulator of BAT that promotes *Ucp1* expression, BAT mass and BAT oxygen consumption, showing increased expression during brown adipocyte differentiation	*Pde1b*	[99]: Mouse; in vitro
MiR-133	Negative regulator of brown adipogenesis, acting as an inhibitor of BAT differentiation	*Prdm16*	[100]: Mouse; in vitro [101]: Mouse; in vitro [102]: In vitro
MiR-155	Negative regulator of brown adipogenesis, enriched in BAT and highly expressed in proliferating brown pre-adipocytes, exhibiting declining expression with cell differentiation	*C/ebpb*	[103]: Mouse; in vitro [104]: Mouse; in vitro [105]: Human; mouse; in vitro
MiR-32	Positive regulator of brown adipogenesis with significantly increased expression in BAT during cold exposure	*Tob1*	[106]: Mouse; in vitro

* All abbreviations are detailed in the list of abbreviations of the manuscript.

**Table 2:** Selected key microRNAs (miRNAs) and their reported effects in brite (beige or brown-in-white) adipose tissue.

miRNA	Function(s)	Target(s) *	Reference(s) and Corresponding Study Model(s)
MiR-196a	Plays a role in browning of white progenitor cells, inducing WAT browning and involved in regulating human body fat distribution	*Hoxc8*	[78]: Mouse; in vitro
MiR-26	Key regulator of human white and beige adipocyte differentiation and positive regulator of brown adipogenesis	*ADAM17, Fbxl19*	[123]: Human; in vitro [124]: Mouse; in vitro
MiR-125	Negative regulator of browning and the formation of functional brite adipocytes	*MMP11*	[125]: Human; in vitro
MiR-Let7i-5p	Thermogenesis inhibitor, strongly inhibiting mitochondrial and browning markers in mice scWAT and human and murine brite adipocytes	Not specified	[126]: Human; mouse; in vitro
MiR-455	Positive regulator of brown/beige AT	*Ucp1*, *Runx1t1,* Necdin, *Hif1an*, *Tgfbr3*	[90]: Human; mouse; in vitro [91]: In vitro [92]: Mouse
MiR-30b/c	Positive regulator of brown and brite adipogenesis	*Rip140*	[93]: Mouse; in vitro [94]: In vitro
MiR-34a	Negative regulator of brown and brite adipogenesis, exhibiting increased expression in obesity, disrupts FGF21 signaling in AT and prevents PGC1A activation and browning of WAT	*Fgfr1*	[95]: Mouse; in vitro
MiR-27b	Inhibitor of brown and beige adipogenesis, downregulated during WAT and brite differentiation	*Prmd16*, *Ppara*, *Pparg*, Creb, *Pgc1b*, Prohibitin	[96]: In vitro [97]: Mouse; in vitro [98]: In vitro
MiR-378	Negative regulator of brite adipogenesis	*Pde1b*	[99]: Mouse; in vitro
MiR-133	Negative regulator of brown adipogenesis	*Prmd16*	[100]: Mouse; in vitro [101]: Mouse; in vitro [102]: In vitro
MiR-155	Negative regulator of brown adipogenesis	*C/ebpb*	[103]: Mouse; in vitro [104]: Mouse; in vitro [105]: Human, Mouse; in vitro
MiR-32	Positive regulator of brown adipogenesis, promoting thermogenesis and WAT browning	*Tob1*	[106]: Mouse; in vitro

* All abbreviations are detailed in the list of abbreviations of the manuscript.

**Table 3:** Selected key microRNAs (miRNAs) and their reported effects in white adipose tissue (WAT).

miRNA	Function(s)	Target(s) *	Reference(s) and Corresponding Study Model(s)
MiR-181	Elevated expression in obese WAT, promotes insulin resistance and inflammation in WAT	*PTEN, TNFA*	[150]: Piglet; in vitro [151]: Review [152]: Human; mouse
MiR-30a	Stimulates adipogenesis, protects adipocytes against inflammation, prevents polarization of macrophages to the M1 phenotype	*STAT1, DLL4*	[94]: In vitro [153]: Mouse; in vitro [113]: Human; mouse
MiR-143	Promotes adipocyte differentiation and insulin resistance	*ERK5, FGF7, MAP3K7, IGF2R, Opr8*	[154]: Rat; in vitro [155]: In vitro [156]: Mouse; in vitro [157]: Human; mouse, in vitro [158]: Human; in vitro
MiR-103	Pro-adipogenic, increases lipid accumulation, attenuates insulin signaling and promotes apoptosis in pre-adipocytes	*RAI14,* Caveolin-1, *Wnt3a*	[159]: Piglet; in vitro [160]: Human; mouse; in vitro [161]: In vitro
MiR-107	Anti-adipogenic, attenuates insulin signaling and promotes apoptosis in pre-adipocytes	*CDK6,* Caveolin-1, *Wnt3a*	[83]: In vitro [160]: Human; mouse; in vitro [161]: In vitro
MiR-221	Negative regulator of adipogenesis, pro-inflammatory effects	*ETS1, Sirt1, AdipoR1*	[162]: Human; in vitro [163]: Mouse; in vitro
MiR-17	Prevents macrophage-mediated AT inflammation and improves insulin resistance	*Ask1*, *STAT3*	[164]: Mouse; in vitro [165]: Mouse; in vitro
MiR-27a	Anti-adipogenic, upregulated expression in 3T3-L1 adipocytes and *ob/ob* mice, increased in the circulation of patients with T2DM or obesity	*Pparg*	[166]: In vitro [167]: Rat; in vitro
MiR-130	Anti-adipogenic, mediates the inhibitory effects of TNFA on PPARG, pro-inflammatory effects	*PPARG, Apcdd1*	[168]: Mouse; in vitro [169]: Human; in vitro [170]: Human; mouse; in vitro
MiR-33	Attenuates adipogenesis and lipid accumulation, regulates cholesterol efflux and HDL synthesis	*HMGA2, ABCA1*	[171]: Mouse; in vitro [172]: Monkey; in vitro [173]: Human; in vitro
MiR-369-5p	Anti-adipogenic	*FABP4*	[174]: Human; in vitro

* All abbreviations are detailed in the list of abbreviations of the manuscript.

## Abbreviations

3’ UTR	3’ untranslated regions
ABCA1	Adenosine triphosphate–binding cassette transporter
ADAM	A disintegrin and metalloprotease domain
ADAM17	ADAM metallopeptidase domain 17
ADIPOQ	Adiponectin gene
AdipoR1	Adiponectin receptor 1
AKT	Protein-kinase B
AMPKα1	AMP-activated protein kinase alpha1
AP2	Adipocyte protein 2
APCDD1	Adenomatosis polyposis coli downregulated 1
ASK1	Apoptosis signal-regulating kinase 1
AT	Adipose tissue
ATF2	Activating transcription factor 2
ATF6	Activating transcription factor 6
ATG7	Autophagy-related gene 7
ATP	Adenosine triphosphate
Bace1	Beta-site amyloid precursor protein cleaving enzyme 1
BAT	Brown adipose tissue
BMI	Body mass index
BMP7	Bone morphogenetic protein 7
C/EBP	CCAAT/enhancer-binding protein
CAD	Coronary artery disease
cAMP	Cyclic adenosine 3′,5′-monophosphate
Cav-1	Caveolin-1
Cdc34	Cell division cycle 34
CDK6	Cell division protein kinase 6
CIDEA	Cell death-inducing DNA fragmentation factor-like effector A
Creb	cAMP-response element binding protein
CVD	Cardiovascular disease
DGCR8	DiGeorge syndrome critical region 8
DLL4	Delta-like protein 4
E2f6	E2F transcription factor 6
EBF2	Early B-cell factor transcription factor 2
Egr1	Early growth factor response 1
EHMT1	Euchromatic Histone Lysine Methyltransferase 1
ER	Endoplasmic reticulum
ERK	Extracellular-signal-regulated kinase
ETS1	E26 transformation–specific-1
FABP4	Fatty acid-binding protein 4
Fbxl19	F-box and leucine rich repeat protein 19
FFA	Free fatty acids
FFAR4	Free fatty acid receptor 4
FGF7	Fibroblast growth factor 7
FGFR1	Fibroblast growth factor receptor 1
FNDC5	Fibronectin type III domain containing 5
GLUT4	Glucose Transporter Type 4
HDAC3	Histone deacetylase 3
HFD	High-fat diet
HIF1an	Hypoxia inducible factor 1 subunit alpha inhibitor
hMADS	Human multipotent adipose-derived stem
HMGA2	High-mobility group AT-hook 2
HOX	Homeobox
HOXC8	Homeobox C8
IgF2	Insulin like growth factor 2
Igf2bp1	Insulin-like growth factor 2 mRNA-binding protein 1
IGF2R	Insulin-like growth factor 2 receptor
Insig1	Insulin-induced gene 1
IR	Insulin resistance
IRS-1	Insulin receptor substrate 1
MAP3K7	Mitogen-activated protein kinase kinase kinase 7
MAPK	Mitogen-activated protein kinase
Mef2	Myocyte enhancer factor-2
MMP11	Matrix metalloproteinase 11
MiR-	MicroRNA
miRNA	MicroRNA
mRNA	Messenger RNA
MYF5+	Myogenic factor 5 positive cells
NAD+	Nicotinamide adenine dinucleotide
NAFLD	Non-alcoholic fatty liver disease
NFKB	Nuclear factor kappa B
OPR8	Oxysterol-binding protein-related protein 8
Pde1b	Phosphodiesterase 1b
Pdgfr2	Platelet-derived growth factor receptor 2
PGC1A	Peroxisome proliferator-activated receptor gamma coactivator 1-alpha
PGC1B	Peroxisome proliferator-activated receptor gamma, coactivator 1-beta
PI3K	Phosphatidylinositol 3-kinase
PKA	Protein kinase cAMP dependent
PNPLA2	Patatin-like phospholipase domain-containing protein 2
PPAR	Peroxisome proliferator-activated receptor
PRDM16	PR domain-containing protein 16
pri-miRNA	Primary miRNA
PTEN	Phosphatase and tensin homolog deleted on chromosome 10
RAI14	Retinoic acid-induced protein 14
RIP140	Receptor-interacting protein 140
RISC	RNA-induced silencing complex
RUNX1t1	Runt-related transcription factor 1; translocated to, 1
SCF	Skp–Cullin–F-box-containing
Sc	Subcutaneous
ScAT	Subcutaneous adipose tissue
SIRT1	Sirtuin-1
SNS	Sympathetic nervous system
SREBPs	Sterol regulatory element-binding proteins
STAT1	Signal transducer and activator of transcription 1
SVF	Stromal vascular fraction
T2DM	Type 2 diabetes mellitus
TBX1	T-Box Transcription Factor 1
TGFB	Transforming growth factor-beta
TgfBr3	Type III transforming growth factor-β receptor
Th1	T-helper 1
TMEM26	Transmembrane protein 26
TNFRSF9	Tumour necrosis factor receptor superfamily member 9
TNFA	Tumour necrosis factor alpha
TOB1	Transducer of erbB-2 1
UCP1	Uncoupling protein 1
WAT	White adipose tissue
